# Exfoliation of the Bones of the Pelvis

**Published:** 1829-05

**Authors:** 


### HOSPITAL REPORTS,

(*Principally condensed from various Periodical Publications.*)

### EXFOLIATION OF THE BONES OF THE PELVIS.

*Case of Exfoliation from the Bones of the Pelvis, causing obstinate Sinuses.** (ROYAL INFIRMARY, EDINBURGH.)

THE object of the paper from which we select the following case is to show that sinuses in the region of the pelvis sometimes depend not on caries, but on death of the bone, which, exfoliating in some part of the pelvis far from the surface, causes continued irritation by the presence of the loose portion; whence it is proper, Mr. SYME observes, in the treatment of all sinuses in this part of the body, not obviously proceeding from caries, to search for such exfoliations, and remove them if they are found to exist.

CASE.—Ninian Mackenzie, aged twenty-two, a plasterer, in the beginning of November last, asked my opinion as to a complaint which he firmly believed to be incurable. He showed me an opening in the left groin, from which there issued a thin gleety discharge, and around which there were many long cicatrices extending all the way from the pubis to the spinous process of the ilium. He also complained of a painful hardness in the lumbar region of the same side, midway between the last rib and crest of the ilium. There was no external tumor, but a distinct induration could be perceived on pressure, which was very painful. In addition to these complaints, he mentioned that his legs were so weak as to prevent him from walking steadily, and that he had frequent desire to make water, with uneasiness in doing so. On desiring to know the history of the case, he gave me the following relation:

Five years ago the scaffold on which he was working happening to give way, he fell with it to the ground, and received in the fall a blow from one of the planks on his left loin. He felt little inconvenience at the time, and continued at the work in which he was engaged; but in the course of a fortnight he began to feel pain in the part struck, which gradually increased and extended into the groin, where a tumor about the size of an egg at length appeared, and induced him to enter the Royal Infirmary of this city two months after the accident. Leeches and other measures of a similar nature were employed, with the effect of removing the tumor, but not the pain. At the end of eight days he returned home, but found himself unable to work for the following fourteen weeks. He then began to do so, when the pain, which had never

* From a paper by JAMES SYME, Esq. Surgeon and Lecturer on Surgery in Edinburgh. Edinburgh Med. Journal, April 1829.

entirely left him, increased in severity, and in the course of two months became very distressing. At the same time the tumor again appeared in his groin, and he now perceived that his left thigh was drawn up to the body, so that he could not extend it. The swelling then opened spontaneously, and discharged an immense quantity of matter, with great relief to all his uneasy feelings; but finding that the running continued for five weeks without any abatement, he once more repaired to the Royal Infirmary, where the sinus was injected, and very freely dilated in the groin, so as to occasion the extensive cicatrices already mentioned. At the end of two months he was dismissed incurable. He went home, and during the five succeeding months was treated by different practitioners of eminence in this city without success: indeed, the means they employed were the same as those found unavailing in the infirmary, viz. injections. He at last concluded the disease to be hopeless, and abstained from all further surgical treatment, working at his trade when the pain, &c. allowed him to do so.

This story, together with my own observations, led me at once to conclude that the painful hardness of the loins depended on an abscess caused by, and containing, an exfoliation of bone; and that, if this source of irritation were removed, as the patient was a stoutly made young man, he would soon get well.

Having explained to him my views of the case, I obtained his ready assent to any thing I might think proper for affording him a chance of recovery, of which he was naturally very desirous, not only on his own account, but on that of his wife and family, who depended on his exertions for their support.

In the presence of my friends, Drs. Mackintosh and Ballingall, I made an incision about three inches long in the left lumbar region, parallel with the crest of the ilium, and, cutting down to the induration, opened an abscess containing a thin fluid. I then introduced my finger, and finding an aperture through the abdominal muscles, searched for the exfoliation, which I soon detected lying on the inner concave side of the ilium, and easily removed by means of a pair of long forceps. Many large sinuses could be felt running in various directions, but not being able to discover any more loose bones, I concluded that every thing necessary had been done, and therefore dressed the wound.

The patient suffered no inconvenience in the way of constitutional disturbance, but a very copious discharge issued from both orifices for several days; it then grew thick, diminished, and ceased at the artificial aperture. It still continued, however, at the old opening; and, as I found that the sinus descended into the thigh somewhat lower than the orifice in question, I dilated it downwards, after which it also soon healed; and on the third week from the operation I showed Mackenzie to my class perfectly well, without any pain or uneasiness of any kind, any defect in his power of progressive motion, or any disturbance of his urinary organs.

